# Serum Prolactin Levels as a Novel, Practical Marker for Predicting Malignant Diseases of the Breast

**DOI:** 10.7759/cureus.58375

**Published:** 2024-04-16

**Authors:** Ikraj Singh, Samir Gupta, Madhura Deshmukh, Madhura Gandhi, Priyanka Khopkar-Kale

**Affiliations:** 1 Surgical Oncology, IMTRAT, Haa Dzong, BTN; 2 Surgical Oncology, Dr. D. Y. Patil Medical College and Hospital, Pune, IND; 3 Central Research Facility, Dr. D. Y. Patil Medical College and Hospital, Pune, IND

**Keywords:** breast cancer, carcinoma of the breast, malignancy, post-menopause, pre-menopause, serum prolactin

## Abstract

Background and objective

Prolactin (PRL) has a high specificity toward breast cancer (BC), making it a valuable marker in both diagnosis and prognosis. In this study, we aimed to compare serum PRL levels between pre- and post-menopausal women with BC, as well as normal reference values. We also investigated the association of various risk factors with PRL levels in women with BC.

Methods

The study involved adult women diagnosed with BC based on clinical features and tissue histopathology receiving treatment at a tertiary care center in Pune, India. General and demographic information, anthropometric measurements (height, weight, and BMI), menstrual status (age at menarche and menopausal state), clinical presentation (signs and symptoms), duration of symptoms, and parity were recorded by using a pre-tested proforma based on hospital records or in-person interviews. Serum PRL was measured by the RIA method (sandwich assay).

Results

A total of 67 women (average age: 47.5 ± 11.8 years; 33 of them post-menopausal) with BC were included in the study. The participants had an average BMI of 24.9 ± 3.5 kg/m^2^,^ ^and 26 (39%) of them were overweight. The majority of women had BC stage IIA disease, involvement of the right side or upper outer quadrant, and had attained menarche after 14 years of age; 47 women had a BC duration of >3 months. Seven women were nulliparous, and the remaining had given birth to their first child before the age of 26 years. The average serum PRL level among the participants was 9.27 ± 7.62 ng/mL, with higher levels found in post-menopausal women compared to pre-menopausal women (11.08 vs. 7.51 ng/mL, respectively; p=0.08). Women with a higher stage and greater duration of disease had significantly higher serum PRL levels (p<0.001 for both). When compared with reference values, pre-menopausal women showed significantly lower (6.25 vs. 10.9, respectively; p=0.001) and post-menopausal women showed significantly higher (8.55 vs. 5.95; p=0.004) serum PRL levels. A positive correlation was found between serum PRL and age at the time of birth of the first child (p=0.068).

Conclusions

Based on our findings, PRL is an important hormone in the development of BC in women. Therapeutic modulation of PRL may be a realistic and novel approach to curing human BC, either administered alone or in combination with conventional treatments.

## Introduction

Breast cancer (BC) is the most common cancer among women globally by far. While it occurs in women at any age after puberty, it is more prevalent later in life. In 2020, there were 2.3 million women diagnosed with BC and 685,000 deaths globally [1]. As per Globocan 2020 data, BC accounted for 13.5% (178,361) of all cancer cases and 10.6% (n=90,408) of all deaths in India, with a cumulative risk score of 2.81 [2]. The total number of cases and net mortality are high in India due to inadequate screening programs and a lack of awareness about BC [3].

BC is more common in nulliparous women and obese post-menopausal women. Both breastfeeding and having the first child at an early age are protective against BC. Recent studies have clarified the role of exogenous hormones in the development of BC, especially oral contraceptive pills and hormone replacement theory [4]. The role of prolactin (PRL) in the pathogenesis and progression of human BC at the cellular, transgenic, and epidemiological levels has been increasingly emphasized. Acting at the endocrine and anticrine/paracrine levels, PRL functions to stimulate the growth and motility of human BC cells [5]. Elevated levels of PRL are related to BC [6,7]. The expression of both PRL and its receptor in human cancer cell lines of diverse origins lends further support to its action as an autocrine/paracrine growth factor [8]. PRL has a high specificity for BC, especially the metastatic variant, making it important for both the diagnosis and the prognosis of this disease [9].

In this study, we aimed to compare serum PRL levels in pre- and post-menopausal women diagnosed with malignant diseases of the breast, as well as the normal reference values for the respective stages. We also endeavored to determine the associations of various risk factors with PRL levels in women with BC.

## Materials and methods

This prospective observational study was carried out at a tertiary care center in Pune, India, from November 2016 to October 2018. Women attending the Surgery Outpatient Department (OPD) were screened based on predefined inclusion and exclusion criteria. Those who were <18 years of age, pregnant, breastfeeding women, those suffering from hormonal disorders of the pituitary or benign diseases of the breast, and those who were on medications that could affect PRL levels were excluded from the study. All cases of malignant diseases of the breast and those willing to participate were included in the study. The study was approved by the Institutional Ethics Committee, and all participants signed an informed consent form.

Diagnosis of BC was established based on clinical features and tissue histopathology. The participants’ general and demographic data were recorded. Anthropometric measurements (height and weight) were performed using calibrated instruments, and BMI was calculated by the formula BMI = weight (kg)/height (m^2^). Menstrual status (age at menarche), clinical presentation (signs and symptoms), duration of symptoms, and parity were recorded from hospital records or by in-person interviews in a pre-tested proforma. Women were considered to be in the pre-menopausal/perimenopausal state if their periods had not ceased, they had a hysterectomy with at least one ovary remaining, and were ≤47 years old (nonsmokers), or were ≤45 years old (smokers). Women were considered in the post-menopausal state if they had not had a period in 12 conservative months, had a bilateral oophorectomy, were ≥56 years old (nonsmokers), or were ≥54 years old (smokers).

Routine and special investigations were performed for each patient to assess their general status and evidence of distant metastasis. Staging of the disease was done according to the American Joint Committee on Cancer (AJCC) Cancer Staging Manual [10]. Venous blood samples were collected from the participants while they were in a state of fasting for the measurement of PRL. To prevent any major variation in the serum PRL levels, samples were collected in the fasting state and before undergoing any breast examination.

After the addition of the reagent, the blood samples were incubated for one hour at 18-25 °C with shaking (>350 rpm). The supernatant was analyzed for serum PRL levels with a kit provided by Beckman Coulter according to the IRMA sandwich assay method. Reference values of 10.9 ng/mL and 5.95 ng/mL were considered the upper limits for pre- and post-menopausal status, respectively [11]. The reference values for serum PRL were obtained from the indicative values of healthy subjects from the kit reference being utilized in the laboratory of the study center. These values align closely with the reference values observed in the Indian population. Tissue diagnosis was done by histopathological examination of the core needle biopsy or excised samples. The disease was staged according to the Union for International Cancer Control (UICC) TNM(p) classification [12].

Statistical analysis was performed using MS Excel (Microsoft 365), RStudio (version 2023.03.1+446), and IBM SPSS Statistics 27. Data are presented using both descriptive and inferential statistics. For quantitative data, mean and standard deviation (SD) or median and interquartile range (IQR) were calculated as appropriate. Normality was checked by using the Shapiro-Wilk test. As the normality assumption was not satisfied and data were skewed, group differences and references were analyzed using the one- or two-sample Wilcoxon rank-sum test. The independent samples Kruskal-Wallis test was used to compare the means of dependent variables within categories of one or more independent variables. Moreover, Pearson’s correlation coefficient was used to determine any association between any two variables. Qualitative variables are expressed as frequencies (percentages). The two-sample proportionality test was applied for comparison between pre-menopausal and post-menopausal groups. For all tests, a p-value <0.05 (two-tailed) was considered statistically significant.

## Results

A total of 67 women with BC were enrolled in the study. The average age of the participants was 47.5 ± 11.8 years, and 28% fell within the age group of 41-50 years. Nearly half (n=33) of the participants were in the post-menopausal state. The average BMI was 24.9 ± 3.5 kg/m^2^, with the majority of women (n=36, 54%) having a normal BMI; only five (7%) women were obese, and 26 (39%) were overweight. There was no significant difference in the anthropometric (i.e., height, weight, and BMI) characteristics between pre- and post-menopausal women. Of note, 47 women had a BC duration of >3 months (Table 1).

**Table 1:** Demographic and anthropometric characteristics of the study participants ^*^P<0.05 indicates statistical significance BC: breast cancer; BMI: body mass index; SD: standard deviation

Characteristic	Total (n=67)	Pre-menopausal (n=34)	Post-menopausal (n=33)	P-value
Age (years), mean ± SD	47.5 ± 11.8	38.0 ± 7.3	57.3 ± 6.2	<0.001^*^
Height (cm), mean ± SD	155.6 ± 4.7	155.7 ± 4.6	155.4 ± 4.8	0.81
Weight (kg), mean ± SD	60.4 ± 8.6	60.5 ± 8.1	60.4 ± 9.2	0.983
BMI (kg/m^2^), mean ± SD	24.9 ± 3.5	24.9 ± 2.9	25.0 ± 4.1	0.861
Obesity gradings, n (%)				
Normal (18.50–24.99 kg/m^2^)	36 (53.7%)	19 (55.9%)	17 (51.5%)	0.91
Overweight (25.00–29.99 kg/m^2^)	5 (7.5%)	1 (2.9%)	4 (12.1%)	0.335
Obese (≥30.00 kg/m^2^)	26 (38.8%)	14 (41.2%)	12 (36.4%)	0.878
Duration of BC (months)	n (%)	n (%)	n (%)	n (%)
<3	20 (29.9%)	11 (32.3%)	9 (27.3%)	0.8514
3–6	24 (35.8%)	14 (41.2%)	10 (30.3%)	0.501
6–12	17 (25.4%)	6 (17.7%)	11 (33.3%)	0.232
>12	6 (9%)	3 (8.8%)	3 (9.1%)	1

The physical and clinical evaluation of the participants revealed the presence of symptoms including lump (n=63, 94.0%), pain (n=31, 46.3%), ulcer (n=16, 23.9%), retraction (n=13, 19.4%), and discharge (n=3, 4.5%). When presenting to the OPD, 34 (50.7%) had right-side involvement, and 49% had upper outer quadrant (UOQ) involvement. At the time of enrollment, 23 (34%) participants had stage IIA BC, and only three (4%) had advanced (IIIC) BC (Table 2). No significant differences between the two groups were observed regarding any of the above-mentioned parameters.

**Table 2:** Physical and clinical examinations of the study participants BC: breast cancer; LIQ: lower inner quadrant; LOQ: lower outer quadrant; UIQ: upper inner quadrant; UOQ: upper outer quadrant

Examination	Total (n=67), n (%)	Pre-menopausal (n=34), n (%)	Post-menopausal (n=33), n (%)	P-value
Symptom				
Lump	63 (94%)	32 (94.1%)	31 (93.9%)	1.000
Pain	31 (46.3%)	17 (50%)	14 (42.4%)	0.627
Ulcer	16 (23.9%)	6 (17.6%)	10 (30.3%)	0.262
Discharge	3 (4.5%)	2 (5.9%)	1 (3%)	1.000
Retraction	13 (19.4%)	4 (11.8%)	9 (27.3%)	0.132
Side involved				
Right	34 (50.7)	21 (61.8%)	13 (39.4%)	0.113
Left	33 (49.3%)	13 (38.2%)	20 (60.6%)	0.113
Quadrant involved				
Central	2 (3%)	1 (2.9%)	1 (3%)	1.000
LIQ	7 (10.4%)	4 (11.8%)	3 (9.1%)	1.000
LOQ	12 (17.9%)	7 (20.6%)	5 (15.2%)	0.794
UIQ	13 (19.4%)	5 (14.7%)	8 (24.2%)	0.497
UOQ	33 (49.3%)	17 (50%)	16 (48.5%)	1.000
BC stage				
IA	1 (1.5%)	1 (2.9%)	0 (0%)	1.000
IIA	23 (34.3%)	13 (38.2%)	10 (30.3%)	0.670
IIB	20 (29.9%)	11 (32.4%)	9 (27.3%)	0.851
IIIA	14 (20.9%)	6 (17.6%)	8 (24.2%)	0.716
IIIB	6 (9%)	1 (2.9%)	5 (15.2%)	0.186
IIIC	3 (4.5%)	2 (5.9%)	1 (3%)	1.000

Patient histories revealed that the majority (n=53) had their menarche after 14 years of age, while the rest had it before 14 years of age. At the time of the study, seven women were nulliparous, and the remaining 60 had given birth to their first child at a relatively younger (i.e., <26 years old).

Of the 67 cases studied, three (4.5%) had ductal carcinoma in situ, 61 (91.0%) had invasive ductal carcinoma, and three (4.5%) had other tissue diagnoses (one with invasive papillary carcinoma, one with medullary carcinoma, and one with mucinous carcinoma) (Table 3). No significant differences were observed between both groups regarding the above-mentioned data.

**Table 3:** Menstrual and obstetric history of the study participants DCIS: ductal carcinoma in situ; IDC: invasive ductal carcinoma; NA: not applicable

Characteristic	Total (n=67), n (%)	Pre-menopausal (n=34), n (%)	Post-menopausal (n=33), n (%)	P-value
Age at menarche (years)				
12–13	14 (20.9%)	9 (26.5%)	5 (15.1%)	0.402
14–15	47 (70.1%)	21 (61.8%)	26 (78.8%)	0.209
>15	6 (9%)	4 (11.8%)	2 (6.1%)	0.697
Parity	n (%)	n (%)	n (%)	
Nulliparous	7 (10.4%)	6 (17.6%)	1 (3%)	0.120
Multiparous	60 (89.4%)	28 (82.4%)	32 (97%)	0.120
Age at first childbirth (years)				
NA	7 (10.4%)	6 (17.6%)	1 (3%)	-
<21	12 (17.9%)	6 (17.6%)	6 (18.2%)	1.000
21–25	46 (68.7%)	22 (64.7%)	24 (72.7%)	0.657
>25	2 (3%)	0 (0.0%)	2 (6.1%)	0.460
Tissue diagnosis				
DCIS	3 (4.5%)	0 (0.0%)	3 (9.1%)	0.227
IDC	61 (91%)	33 (97.1%)	28 (84.8%)	0.186
Other	3 (4.5%)	1 (2.9%)	2 (6.1%)	0.979

**Figure 1:**
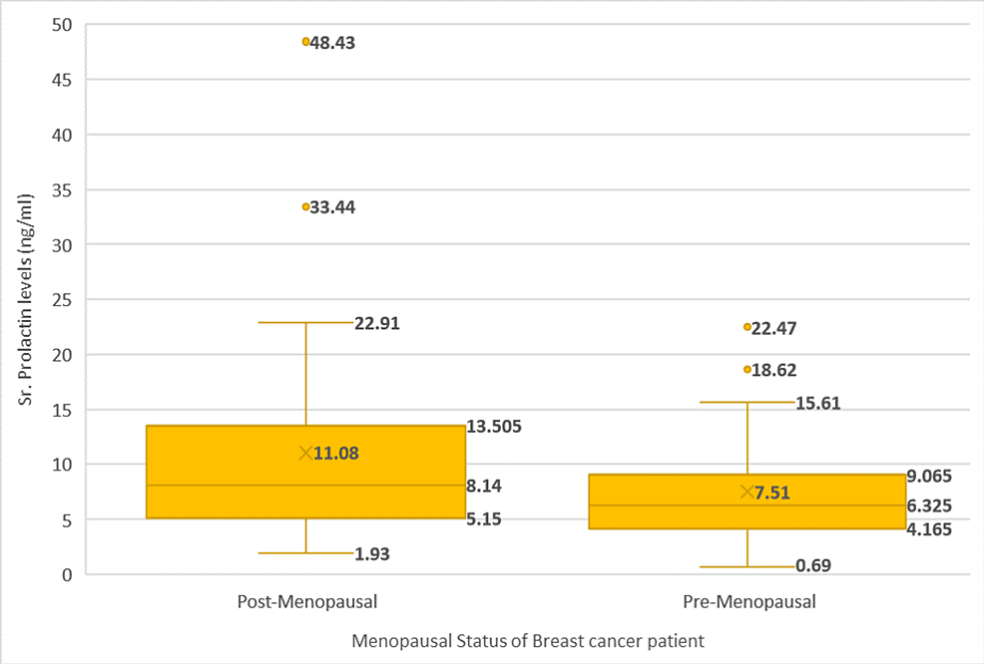
Comparison of serum prolactin levels in breast cancer patients of different menopausal status Values are displayed as means ± standard deviations. Values represent three quartiles; mean, range

The patients had an average serum PRL level of 9.27 ± 7.62 ng/mL. Post-menopausal women had higher serum PRL levels compared to pre-menopausal women, with borderline statistical significance (11.08 vs. 7.51 ng/mL, respectively; p=0.08), as seen in Figure 1.

Serum PRL levels were significantly higher in those with higher stages or greater durations of the disease (p<0.001 for both). The pre-menopausal group showed significantly lower levels of serum PRL (6.25 vs. 10.9, respectively; p=0.001) as compared to the provided reference values, whereas the post-menopausal group showed significantly higher levels (8.55 vs. 5.95, respectively; p=0.004), as seen in Tables 4-5.

**Table 4:** Serum prolactin levels in relation to breast cancer stage of the participants ^*^P<0.05 indicates statistical significance BC: breast cancer; IQR: interquartile range; SD: standard deviation

Characteristic	Serum prolactin levels (ng/mL)			P-value
Stage of BC	n (%)	Mean ± SD	Median (IQR)	
IA	1 (1.5%)	2.61	2.61	<0.001^*^
IIA	23 (34.3%)	5.52 ± 3.32	4.63 (3.64–7.15)	
IIB	20 (29.9%)	7.22 ± 2.97	6.34 (4.93–8.59)	
IIIA	14 (20.9%)	11.26 ± 6.9	9.87 (7.67–11.95)	
IIIB	6 (9%)	16.65 ± 5.99	17.85 (10.53–22.08)	
IIIC	3 (4.5%)	29.84 ± 16.21	22.47	
Duration of disease (months)				
<1	4 (6%)	6.28 ± 1.67	6.31 (4.71–7.81)	<0.001^*^
1 to 3	16 (23.9%)	6.84 ± 3.40	6.83 (4.24–8.5)	
3 to 6	24 (35.8%)	5.81 ± 3.37	4.94 (3.75–6.61)	
6 to 12	17 (25.4%)	12.68 ± 7.27	10.63 (8.06–15.13)	
>12	6 (9%)	21.92 ± 13.95	19.36 (12.32–28.96)	

**Table 5:** Serum prolactin levels in relation to reference values ^*^P<0.05 indicates statistical significance CI: confidence interval; SD: standard deviation

Status	Mean ± SD	Reference value	P-value	95% CI
Pre-menopausal (n=34)	7.51 ± 4.67	10.9	<0.001^*^	(5.42, 8.34)
Post-menopausal (n=33)	11.08 ± 9.52	5.95	0.002^*^	(7.05, 12.3)

When we investigated the correlation between risk factors for BC and serum PRL levels, a positive correlation with borderline significance (p=0.068) was found with age at the time of birth of a first child. However, other risk factors (e.g., age, height, weight, BMI, and age at menarche) were not significantly correlated with serum PRL level (Table 6).

**Table 6:** Correlations between risk factors for breast cancer and serum prolactin levels BMI: body mass index

Risk factor	Pearson's correlation coefficient	P-value
Age	0.164	0.186
Height	−0.13	0.296
Weight	0.128	0.304
BMI	0.192	0.119
Age at menarche	0.107	0.387
Age at the birth of the first child (n=60)	0.238	0.068

## Discussion

In the present study, we reported the demographic, clinical, and biochemical profiles of 67 cases of BC at a tertiary care center. We found that a significant proportion (n=19, 28.4%) of women were between 41 and 50 years of age, with an average age of 47.5 ± 11.8 years. At the time of the study, the duration of carcinoma was >3 months in the majority of women, and half of the participants were in the post-menopausal state. The average serum PRL level of the participants was 9.27 ± 7.62) ng/mL. We found significantly higher levels of serum PRL in post-menopausal women when compared with pre-menopausal women, as well as with reference values. Serum PRL levels were significantly higher in women of advanced age, as well as those with an advanced stage of the disease.

The worldwide distribution of BC indicates that its incidence increases progressively with age, and our findings among Indian women with BC align with those reported by Tworoger et al. and Raina et al., who reported mean ages of 45.3 years and 47 years in their study populations, respectively [13,14]. We also investigated other risk factors for the development of BC in our study population. Age at menarche is one of the main risk factors for BC, as in the present study, where 14 (20.9%) participants had an age at menarche of 12-13 years, 47 (70.1%) were 14-15 years in age, and six (9.0%) were >15 years in age. A similar result was reported in another study, where the mean age at menarche was 12.8 years [15]. Nulliparity has been associated with an increased risk for BC, as observed among nun populations [16]. In the current study, seven (10.4%) participants had nulliparity. McMohan et al. have reported that having a larger number of children decreased the risk of BC; women with four or more children had half the relative risk compared to those with nulliparity [15].

Previous studies have noted that women who become pregnant before 18 years of age and have a full-term pregnancy have a BC risk that is approximately one-third of that of those who become pregnant for the first time after 35 years of age [16-18]. In our study, 17 (10.4%) participants never had any children, 12 (17.9%) were below 20 years of age at the time of the birth of their first child, and 38 (56.7%) were 21-23 years of age at the time of the birth of their first child. When they presented to the OPD, 34 (50.7%) participants had right-side involvement, and the largest group (49%) had UOQ quadrant involvement, which is in line with the findings of other studies [19,20]. The median duration of symptoms was reported to be approximately six months in a study by Thakur et al. [21]. In the present study, 47 participants had a BC duration of >3 months, providing strong evidence that delayed presentation of symptomatic BC is associated with a lower survival rate. The presence of a lump was the most common symptom observed in the study population. Similar results have been reported in the literature [22].

Infiltrating duct carcinoma (91%) was found to be the most common histological variant of BC, similar to observations made by other authors [19,20]. It was evident from this study that most of the tumors belonged to AJCC Stage II, mostly IIA and IIB (34.3% and 29.8%, respectively). These results were similar to those obtained in the study by Mohapatra et al. [23]. The 67 study participants had an average serum PRL level of 7.29 ng/mL, with a statistically significant increasing linear trend in those with a higher stage of the disease. Similar findings were seen in a study conducted by Bhatavdekar et al. [24]. Serum PRL levels were significantly higher in post-menopausal patients compared to the reference value (p<0.01). Similar observations have been made in other studies [24,25], as well as in a meta-analysis by Wang et al. [26]. We found significantly higher levels of serum PRL in the post-menopausal group than in the pre-menopausal group (8.55 ng/mL vs. 6.25 ng/mL). This contrasts with the observations made by Sarfaty et al. and Bani et al., who found higher serum PRL levels in pre-menopausal women as compared to post-menopausal women [27,25].

Elevated PRL levels are often found in metastatic BC patients. In the Nurse’s Health Study and Nurse’s Health Study II, Tvoroger et al. investigated the relationship between PRL and BC risk by measuring PRL levels at <10 and ≥10 years before the diagnosis of BC. After 20 years of follow‐up, they found an association between PRL levels <10 years before diagnosis and BC risk in post-menopausal women, especially in estrogen receptor-positive tumors and metastatic disease [13]. Thus, the detection of raised serum PRL levels can be a useful indicator of high risk for BC. Recently, trials have been conducted with therapy directed at the PRL receptor in the breast, with promising results [28]. We focused on highlighting this correlation between PRL and BC risk. A better understanding of the differential actions of PRL under varied hormonal conditions in pre-menopausal and post-menopausal women and elucidation of biological pathways involved may provide further insights into the changes noticed. In addition, PRL has a strong circadian rhythm [29], increasing after a noontime meal [30]; however, in our study, all samples were carefully collected with the patient fasting, to minimize misclassification.

Our study is limited by its small sample size and absence of a comparator group. Therefore, observational and genetic studies with a larger cohort are required to validate our conclusions and also to investigate possible underlying mechanisms. Phases of the menstrual cycle should have been included in the study analysis to make the results stronger. Additionally, the immunoassay method measured multiple PRL isoforms, which may have different biological activities; however, this was not further explored to identify which isoforms were most prevalent in our case and, thus, most important in terms of BC risk.

## Conclusions

We found significantly higher levels of serum PRL in post-menopausal women when compared with pre-menopausal women, as well as with reference values. Advanced age and advanced stage of the disease were positively associated with serum PRL levels. Based on our findings, PRL is an important hormone in the development of BC in women. Nevertheless, further investigations through larger laboratory and epidemiologic studies are required to substantiate the mechanisms underlying this association. Moreover, therapeutic modulation of PRL may be a realistic, novel approach to curing human BC, either given alone or in combination with conventional treatments.
